# Effect of Long-Term Paddy-Upland Yearly Rotations on Rice (*Oryza sativa*) Yield, Soil Properties, and Bacteria Community Diversity

**DOI:** 10.1100/2012/279641

**Published:** 2012-07-31

**Authors:** Song Chen, Xi Zheng, Dangying Wang, Liping Chen, Chunmei Xu, Xiufu Zhang

**Affiliations:** ^1^China National Rice Research Institute, Chinese Academy of Agricultural Sciences, Zhejiang, Hangzhou 310006, China; ^2^985-Institute of Agrobiology and Environmental Sciences, Zhejiang University, Hangzhou 310029, China

## Abstract

A 10-year-long field trial (between 2001 and 2010) was conducted to investigate the effect of paddy-upland rotation on rice yield, soil properties, and bacteria community diversity. Six types of paddy-upland crop rotations were evaluated: rice-fallow (control; CK), rice-rye grass (RR), rice-potato with rice straw mulches (RP), rice-rapeseed with straw incorporated into soil at flowering (ROF), rice-rapeseed incorporated in soil after harvest (ROM), and rice-Chinese milk vetch (RC). Analysis of terminal restriction fragment length polymorphism (T-RFLP) was used to determine microbial diversity among rotations. Rice yield increased for upland crops planted during the winter. RC had the highest average yield of 7.74 t/ha, followed by RR, RP, ROM, and ROF. Soil quality differences among rotations were found. RC and RP improved the soil mean weight diameter (MWD), which suggested that rice rotated with milk vetch and potato might improve the paddy soil structure. Improved total nitrogen (TN) and soil organic matter (SOM) were also found in RC and RP. The positive relationship between yield and TN/SOM might provide evidence for the effect of RC rotation on rice yield. A strong time dependency of soil bacterial community diversity was also found.

## 1. Introduction

In China and other Asian countries, continuous rice planting has had a negative impact on soil properties, such as reduced soil nitrogen supply and organic carbon content [1, 2]. Paddy-rice-upland crop rotations have been recommended and used to improve soil quality and reduce input [3–8].

In conventional paddy-upland rotation systems, farmers drain the fields after harvesting rice and then plant an upland crop, such as milk vetch, wheat, or oilseed [9–11]; However, the growth conditions required by rice are quite different from those required by upland crops. Rice will grow best under puddled, reduced, and anaerobic soil conditions, whereas upland crops require unpuddled, aerobic and oxidized soil conditions. Paddy soils show a large difference from upland soils in physical, chemical, and biological properties [12]. Furthermore, because of long-term submergence and mineral fertilizer application, paddy soils experience degradation of soil quality, such as breakdown of stable aggregation and deterioration of soil organic matter (SOM), which negatively affects agricultural sustainability [13, 14].

Soil quality is a term used to describe the health of agricultural soils. It has been suggested as an indicator for evaluating sustainability of soil and crop management practices [15–17]. Many soil attributes have been proposed to describe soil quality, but evaluation of pH, soil organic matter (SOM), and total nitrogen content (TN) of soil have been considered essential for assessing the chemical aspects of soil quality [15, 17]. These chemical traits are so important because they provide a measure of the ability of soil to supply nutrients and to buffer against chemical additives [18–20].

Soil physical properties are indicators of the impact of soil and crop management practices. Soil size distribution and water stability of soil aggregates would be influenced by crop types as well as soil management practices [21]. Furthermore, microbial populations in soil interact with each other and with soil. These interactions, in turn, affect major environmental processes, including biogeochemical cycling of nutrients, plant health, and soil quality [22]. Most microbial interactions in soil occur near the plant roots and the root-soil interface, called the rhizosphere [23, 24]. It is not surprising that microbial communities in the rhizosphere depend on plant species [25, 26]. Although the relationships between soil microbial diversity and function and sustainability (or stability) of agricultural ecosystems are still unclear [17, 27, 28], it has been documented that diversity of soil biota is important to the beneficial function of agro-ecosystems [29, 30].

In China, paddy-upland crop rotation is a major cropping system utilized along the Yangtze River basin [31–33]. Traditionally, the main patterns of rice and upland crop are rice-wheat, rice-oilseed, rice- milk vetch, and rice-ryegrass [4]. However, some practices may not be economical because of high input and increased risk of financial loss for the rice crop. Currently, new patterns including rice-oilseed rape (*Brassica napus* L.) and rice-potato (*Solanum tuberosum*) are widely used. However, few studies have been done to determine the effect of these paddy-upland crop rotations on soil physical and chemical properties. The present study was conducted to determine the effect of long-term cropping system on (i) rice and other crop yields, (ii) soil quality attributes (chemical and physical), and (iii) the diversity of soil bacterial community. The results offer helpful insight into the effect of various rice-upland combinations on crop productivity and soil properties. 

## 2. Materials and Methods

### 2.1. Soil and Site

The study was conducted over a period of ten years at the experimental farm of the China Rice Research Institute (120.2 E, 30.3 N, and 11 m above sea level) located in Fuyang, Hangzhou, China. The long-term field experiment has been carried out in an irrigated rice paddy starting in 2001. The historical cropping background was monoculture rice cropping before this time. The area is characterized by a subtropical monsoon climate with an annual mean temperature of 13–20°C, ranging from 2°C in January to 35°C in July and mean precipitation of 1200–1600 mm per year, with about 80% falling between April and September. Soil is classified as Ferric-accumulic Stagnic Anthrosols [34] and entic Halpudept [35]. Further details about the experimental site can be found in the study of Fu et al. [36].

### 2.2. Experimental Design and Crop Cultivation

The long-term field experiment included six different types of cropping rotation: continuous monoculture rice-fallow (CK), rice-ryegrass (*Lolium perenne*) (RR), rice-potato (*Solanum tuberosum*) (RP), rice-Chinese milk vetch (*Astragalus sinicus L.*) (RC), rice-oilseed rape (*Brassica napus L.*) with burned straw returned to the soil after harvest (ROM), and rice-oilseed rape with fresh oilseed straw returned as mulch at flowering (ROF). Primary tillage was followed by a pass of a stubble crushing machine to a depth of 0–20 cm about three days before rice seedling transplanting in early June every year. During the rice season, the rice species used was Guodao 6, an elite *Japonica* hybrid widely planted in Southeast China. Direct sowing was applied for seedling establishment, with a seeding rate of 15 kg/ha. The amount of fertilizer application was set according to local agronomic practices. It is noteworthy that there was a significant reduction in nitrogen input starting in 2005 due to the serious rice lodging of several rotations. The rate and timing of fertilizer was set as follows: basal application, including total phosphorous, 50% nitrogen, and 50% potassium, was conducted a week before transplantation in the form of compound fertilizer; 25% nitrogen was applied as topdressing at mid-tillering in the form of urea, and 25% nitrogen and 50% potassium was applied as topdressing at panicle initiation in the form of urea and potassium chloride, respectively. Weeds, insects, and diseases were controlled as required to avoid yield loss. More details of the agronomic practice are presented in Table 1.

The field experiment was planned with a large plot area (20 m long, 20 m wide) but without treatment replicates due to practical reasons. Although this design could lead to statistical problems, the cropping system has been used for decades with uniform fertilizer management. The small variability (CV < 5%) of the representative parameters of soil fertility in the treatment plots (Table 2) indicates a low spatial heterogeneity of the field. We therefore believe it is reasonable to assume that any significant differences later observed between plots were caused by the different rotations.

### 2.3. Sampling and Measurement

In 2010, a survey of rice yield was carried out as follows: hills were harvested from the uniform part of each plot of 5 m^2^ with 4 subsamples. Unhulled (rough) rice was obtained after reaping, threshing, and wind selection. Rough rice from 80 hills was hulled and then put through a 1.8 mm sieve to remove any immature kernels. The weight of hulled rice (brown rice) was adjusted to a moisture content of 14%. Upland crop biomass was sampled from 1 m^2^ of four sub-sample plots. For RC, RP, ROM, and ROF, plants were sampled at harvest. For RR, sampling was carried on during the growth season. All samples were oven-dried at 70°C to a constant weight to determine the dry weight.

Soil samples from 0 to 20 cm depth at pretransplanting in early June 2010 were used for soil physical quality analysis. Bulk density was determined for undisturbed soil samples using a steel cylinder of 100 cm^3^ volume (5 cm in diameter, and 5.1 cm in height) [37] Soil aggregation was determined by the wet sieving method [38]. The mean weight diameter was calculated as follows:

(1) MWD=∑i=1nXiWi,

where MWD is the mean weight diameter of water-stable aggregates, *Xi* is the mean diameter of each size fraction (mm), and *Wi* is the proportion of the total sample mass in the corresponding size fraction after the mass of stones was deducted (upon dispersion and passing through the same sieve). 

Soil samples for chemical analysis were collected at pretransplanting in early June 2010 from two depths (0–10 and 10–20 cm). The field-moist soil samples were passed through an 8 mm sieve and air-dried. Cation exchange capacity (CEC) was measured according to the procedure used by Hendershot et al. [39]. Electrical conductivity was measured using a digital conductivity meter. Soil total N (TN) and total P were estimated by the methods given by Bremner and Mulvaney [40] and Sparling et al. [41], respectively. Soil water content was determined gravimetrically (105°C, 24 h). Results were expressed based on the dry weight of the soil. Soil pH was determined (1 : 5 water suspension) by pH meter. Soil available potassium was determined by extraction with 1 mol NH_4_AC. Soil organic matter (SOM) was determined by a dichromate oxidation procedure. Multiplying the soil organic carbon by 1.72 resulted in the SOM [42]. The microbial biomass C (MBC) was determined using the chloroform fumigation-extraction method on fresh soils [43]. Each replicate was divided into two equivalent portions: one was fumigated for 24 h with ethanol-free chloroform and the other was not fumigated as a control. Both fumigated and unfumigated soils were shaken for 30 min with 0.5 M K_2_SO_4_ (1 : 4 soil : extraction ratio) and centrifuged and filtered. Extracts were analyzed for DOC on a total organic C analyzer (TOC-V CPH, Shimadzu). 

Soil samples for T-RFLP analysis were collected from each plot using a soil auger (5 cm in diameter) at pretransplanting in early June 2010 (BR) and after harvest in late October 2010 (AR) from 0–20 cm soil depth. Samples were packed in sterile plastic bags and sent to the laboratory, then air-dried until the water content was about 75%. Later, the moist soil was passed through a 2 mm sieve and stored at 4°C for DNA extraction.

### 2.4. DNA Extraction and T-RFLP Analysis

Genomic DNA of the soil samples was isolated using a SDS-hyperhaline buffer solution as used in Zhou et al. [44]. Approximately 1 g of dry soil was suspended in 2.7 mL of extraction buffer (100 mM Tris-HCl, 100 mM EDTA, 100 mM Na_3_PO_4_, 1.5 mM NaCl, 1% CTAB, pH 8.0), proteinase K (20 *μ*L) was added, and the mixture was shaken at 225 rpm at 37°C for 30 min. The suspension was further incubated in 20% SDS at 65°C for 2 h. During incubation, the tubes were gently frozen-thawed with liquid nitrogen for 20 min and run for three cycles. After that, the soil samples were centrifuged at 8,000 rpm for 10 min at 4°C, followed by the extraction of supernatant with 2.6 mL chloroform-isoamyl alcohol (24 : 1) and centrifuged at 5,000 rpm for 5 min. The supernatant was precipitated for 2 h with 2 mL isopropanol before recovering the DNA with 10,000 rpm centrifugation for 10 min. The resulting pellet was washed with 2 mL of 70% (v/v) ice-cold ethanol, dried, and dissolved in 200 *μ*L of sterile distilled water. The purified DNA was stored at 4°C for at least 1 day before PCR amplification. Preliminary analysis showed that the heterogeneity of the profiles obtained from independent preparations of the same soil sample decreased after storing of the fresh DNA. 

The eubacterial primers 8f (5′-AGAGTTTGATCCTGGCTCAG-3′) labeled at the 5' end with 6-carboxyfluorescein (6-FAM) and 926r (5′-CCGTCAATTCCTTTRAGTTT-3′) were used to amplify approximately 920 bp of the 16S rRNA gene [45]. Each PCR reaction mixture (25 *μ*L) contained 2.5 *μ*L 10 × *TransTaq* HiFi Buffer I (200 mM Tris-HCl (pH 8.4), 200 mM KCl, 100 mM (NH_4_)_2_SO_4_, 20 mM MgCl_2_), 2 *μ*L 2.5 mM dNTPs, 2 *μ*L 10 *μ*M primers, 0.5 *μ*L 5 unit *μ*L^−1^ of *TransTaq* polymerase High Fidelity (Beijing TransGen Biotech Co., Ltd. China), and 2 *μ*L genomic DNA temples in a final volume of 25 *μ*L. All amplifications were performed on a DNA Engine Dyad thermal cycler (Bio-Rad, Inc., USA) using the following program: a 5-min hot start at 94°C, followed by 39 cycles consisting of denaturation (1 s at 94°C), annealing (45 s at 50°C and 60°C, resp.), and extension (1 min at 72°C), with a final extension at 72°C for 10 min. PCR products were detected using 1.0% agarose gel electrophoresis in a 1 × TAE buffer. Fluorescently-labeled PCR products (200 *μ*L) were purified with a UNIQ-10 DNA purification kit (Sangon Biotech Co., Ltd., China). Approximately 50 ng of amplified 16S rRNA gene fragments was digested with HaeIII (GG′CC), HhaI (GCG′C), and HinfI (G′ANTC) for 4 h at 37°C and precipitated with isopropanol. The precipitated DNA was washed twice with 70% isopropanol, vacuum dried, and resuspended in 20 mL water. In the preliminary experiments, seven restriction enzymes (HaeIII, HhaI, HinfI, MspI, AluI, and TaqI) were tested; the enzymes HhaI, HinfI, and HaeIII had the higher yield. The sample was mixed with 0.1 mL of GeneScan 1000 Rox size standard, denatured at 95°C for 2 min, immediately placed on ice, and evaluated following electrophoresis in POP6 polymer with an automated DNA sequencer (ABI 3100, Applied Biosystems Instruments, California, USA). Terminal fragment sizes between 29 and 940 bp were determined using Gene-Mapper v3.7 software (Perkin-Elmer, California, USA).

### 2.5. Data Analysis

Data were analyzed by using Microsoft Excel 2003 and SAS 8.0 (2003). Means and standard deviations/standard errors are reported for each of the measurements. One-way analysis of variance (ANOVA) of Tukey's test was used to compare the effects of rotations on soil properties determined for the two soil depths of 0–10 cm and 10–20 cm separately.

All T-RFLP community profiles were labeled for statistical analyses by rotation (CK, RR, RP, RC, ROM, or ROF), sampling time (BR or AR), restriction enzyme (HaeIII, HhaI, or HinfI), and field plot replicate (1, 2, or 3). T-RF peaks between 35 and 500 bp and peak heights of <50 fluorescence units were included in the analysis according to the range of the size marker. Generally, the error for determining fragment sizes with our automated DNA sequencer was less than 1 bp; however, in some cases, a higher variation was found. Therefore, T-RFs that differed by less than 1.5 bp were clustered unless individual peaks were detected in a reproducible manner. Three replicate samples of all rotations and particle sizes were analyzed individually, or a representative sample profile was determined in a way similar to that suggested by Dunbar et al. [46]. Essentially, the sum of the peak heights in each replicate profile was calculated and used to indicate the total DNA quantity. Total fluorescence was adjusted to the medium DNA quantity by calculating a correction factor. For example, three replicate profiles had total fluorescence values of 4,500, 4,700, and 4,900, and then each peak in the latter profile was multiplied by a factor of 0.96 (i.e., the quotient of 4,700/4,900), and peaks in the first profile were multiplied by a factor of 1.04 (i.e., a quotient of 4,700/4,500). After adjustment, only peaks of >50 fluorescence units were considered. In addition, T-RFs were scored as positive only when they were present in at least two of the three replicates.

In order to determine similarities between T-RFLP profiles, a binary matrix that recorded the absence and presence of aligned fragments was generated. The distance matrix of fragments was generated according to the Jaccard index (1908) using NTSYS version 2.10e software for PC (Applied Biostatistics). Based on the distance matrix, cluster analysis was performed utilizing an unweighted pair group method with arithmetic average (UPGMA). 

## 3. Results

### 3.1. Rice Yield and Upland Crops Biomass Production

**Figure 1:**
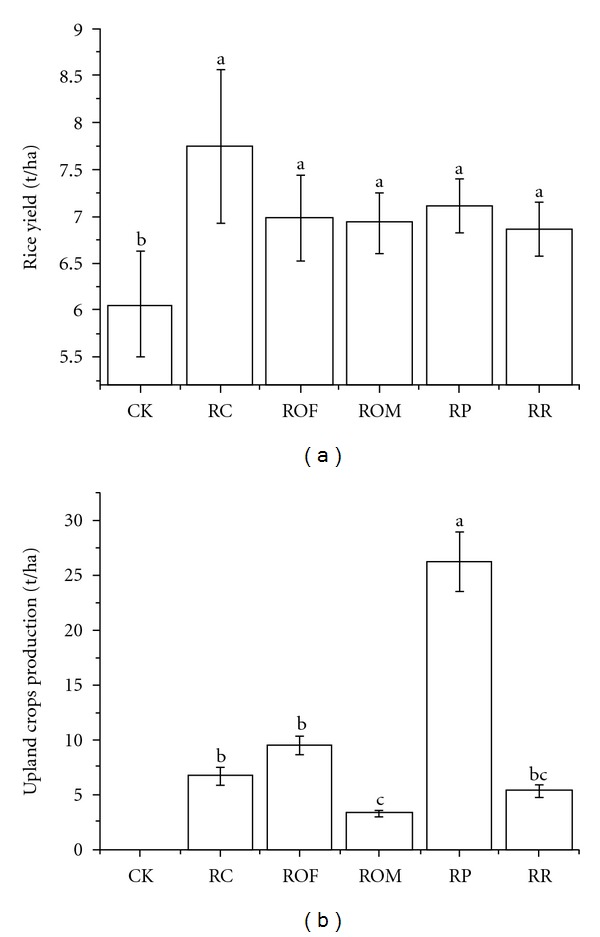
Effect of different paddy-upland crops rotations on the rice yield (a) and upland crops biomass production (b) during 2010-2011. Error bars represent standard deviation, *n* = 3. Values followed by different lowercase letters within depth are significantly different between treatment by Tukey's test (*P* < 0.05). Abbreviations: CK, rice-Fallow phase; RR, rice-ryegrass; RP, rice-potato, with rice straw as mulch; RC, rice-Chinese milk vetch; ROM, rice-oilseed rape with burned straw return after harvest; ROF, rice-oilseed rape with fresh straw return as mulch at flowering.

Rice yield increased in the plots with upland crops applied during the winter season (Figure 1(a)). RC had the highest average yield of 7.74 t/ha, which was 27.8% significantly higher than CK. No significant difference was observed among RR, RP, ROM, and ROF, although their average yields were also higher than CK, with average yield increase ranging from 14.2–17.8%. Upland crop biomass production was estimated and is shown in Figure 1(b). RP had the highest average biomass production of 22.3 t/ha, followed by ROF, RC, RR, and ROM. In CK, weeds grew and died during the fallow phase, making it difficult to estimate the biomass production due to the uneven growth of the weeds. As a result, no data are shown here to describe the biomass production in CK. Different from RP, RC, ROM, and ROF, biomass produced in RR was removed from the field as pasture crops. But all the plant residues in ROF, RP, and RC were returned to the field as cover crops and incorporated into soil before rice season. For ROM, the straw was burned after harvest and the ash was incorporated into soil by tillage. Generally speaking, RP had the highest value of biomass C return, followed by RC and ROF. ROM and RR might have the lowest values next to CK.

### 3.2. Effect of Paddy-Upland Rotation System on Soil Properties

The soil bulk density, soil aggregation, and mean weight diameter (MWD) of different paddy-upland crop rotations are presented in Table 3. The bulk density was significantly greater in RC and ROF than in the others, and RP and RC had relatively higher values of MWD compared with other rotations.

There was a strong depth-dependency of soil pH value, total soil nitrogen (TN), total soil phosphorus (TP), available potassium (K), and cation exchange capacity (CEC) in all rotations (see Table 4). Soil was acid in all six rotations from 0–10 cm and 10–20 cm depth. However, the average pH value was significantly greater in 10–20 cm (5.90) than in 0–10 cm (5.44). In comparison with CK, the pH values in RC, ROF, RP, and RR decreased in 0–10 cm depth, but increased in the 10–20 cm depth for ROF and ROM.

For TN and TP, remarkable differences were found between depths, with 0–10 cm greater than 10–20 cm. The average values of TN/TP were 2.73/0.63 (g/kg) in 0–10 cm and 2.51/0.51 (g/kg) in 10–20 cm. However, the difference of available K between depths was not statistically significant. The rotation effect on soil TN, TP, and available K was significant. In comparison with CK, the values of TN were significantly increased in RC, ROF, and RP in 0–10 cm, with average increments of 18.7%, 14.6%, and 20.3%, respectively. However, similar results in 1–20 cm were only found in RC and RP, with average increments of 10.6% and 14.4%, respectively. There was a variation of rotation effects on TP in 0–10 cm, with ROF (0.70 g/kg), RP (0.72 g/kg), and RR (0.67 g/kg) significantly greater than CK (0.53 g/kg). However, little difference in TP was found in 10–20 cm depth among all rotations. The available K was greater in ROF, ROM, and RP compared with CK in 0–10 cm depth. However, little difference was found among rotations in 10–20 cm depth, except for ROM, which had the highest value among the six rotations. The rotation effect on soil CEC was variable with soil depth. For 0–10 cm depth, CEC in RC and ROM was significantly greater than that in CK, but only RC had significantly different results in 10–20 cm depth.

Soil organic matter (SOM), soil dissolved organic carbon content (DOC), and soil microbial biomass carbon content (MBC) of the six rotations in two soil depths were analyzed and are shown in Table 5. Significant difference was found in SOM between soil depths, with values in 0–10 cm greater than those in 10–20 cm. SOM of rotations ranged from 18.9–25.8 g/kg in 0–10 cm to 16.6–23.4 g/kg in 10–20 cm depth. Moreover, RP rotation was greater than CK in both depths. However, SOM in ROF and ROM decreased compared with CK in both depths. Small differences for DOC and MBC between depths were found, but the difference among rotations was significant. DOC in CK was the lowest among rotations in both depths. Compared with CK, DOC and MBC increased in RC, RP, and ROF, but decreased in ROM and RP.

### 3.3. Diversity of Bacterial Communities in Soils from Different Paddy-Upland Crop Rotation Systems

**Figure 2:**
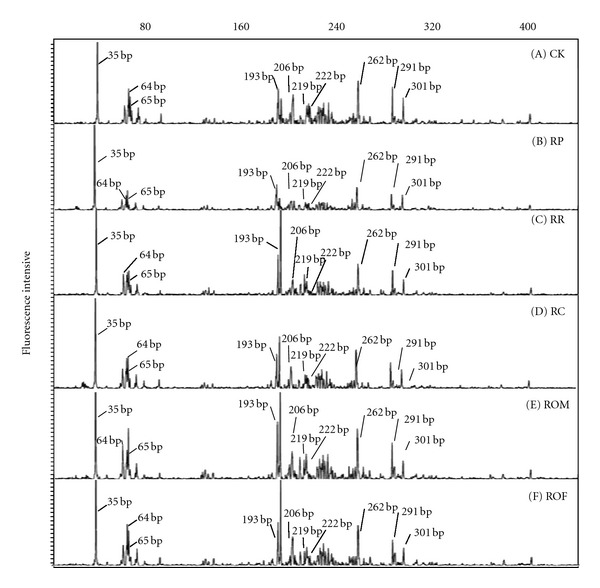
Terminal restriction fragment length polymorphism profiles of soil bacterial communities derived from six different rotation fields before transplanting (BR): CK (rice-fallow phase), RR (rice-ryegrass), RP (rice-potato, with rice straw as mulch), RC (rice-Chinese milk vetch), ROM (rice-oilseed rape with burned straw return after harvest), and ROF (rice-oilseed rape with fresh straw return as mulch at flowering) are shown. Terminal fragments were generated by a HaeIII digestion of 16S rRNA gene fragments amplified from total community DNA. Selected terminal restriction fragments differing in their relative abundance between the studied sites are indicated.

Terminal restriction fragment length polymorphism analysis of 16S rRNA gene fragments amplified from community DNA was applied to compare the bacterial community structure in the field sites described above. Consistent T-RFLP profiles were obtained from three sampling points of the same field site, as shown by respective replications of the six different rotations (Figure 2).

**Figure 3:**
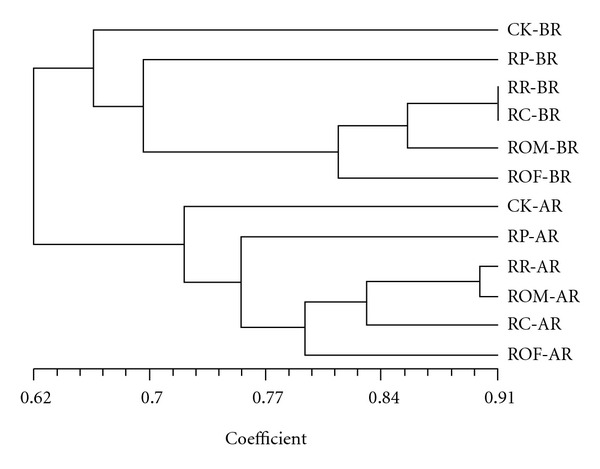
UPGMA dendrogram generated from all representative T-RFLP sample profiles. The scale indicated the coefficient between soil from paddy-upland crop rotation systems sampled at postharvest (AR) and pretransplanting (BR); Abbreviations: CK, rice-fallow phase; RR, rice-ryegrass; RP, rice-potato, with rice straw as mulch; RC, rice-Chinese milk vetch; ROM, rice-oilseed rape with burned straw return after harvest; ROF, rice-oilseed rape with fresh straw return as mulch at flowering.

As shown by cluster analysis, a total of eight rotations formed mainly two major separate branches: pretransplanting (BR) and postharvest (AR) (Figure 3). Within each branch, the lowest similarity was found in CK (followed by RP) regardless of sample time, with a mean similarity to others of 62.5% for BR and 71.3% for AR. Further, four out of the other five rotations were grouped together with a mean similarity to RP of 69.8% for BR and 75.8% for AR. These results indicate remarkable differences in the effect of long-term paddy-upland rotations on soil bacterial community construction.

## 4. Discussion

### 4.1. Paddy-Upland Crop Rotation Effect on Rice Yield and Upland Crop Biomass Production

Rice yield increased when upland crops were applied during the winter season (Figure 1(a)). The increases were slight but positive, indicating the yield benefit of long-term application of paddy-upland crop rotations. Similar results were reported by Ghoshal and Singh [47] and Kim et al. [48]. However, in terms of different agronomic practice, such as upland crop species and the amount of organic and chemical fertilizer input, the magnitudes of these increases were variable. RC had the highest average yield of 7.74 t/ha, which was 27.8% higher than CK. The yield increase might be attributed to the high nitrogen fix capabilities and biomass accumulation in RC [49, 50] However, the capacity of green manure to sufficiently supply soil nutrients is still variable and depends on biomass production and soil management. Slight increases were found in RR, RP, ROM, and ROF compared with CK, although the differences were not statistically significant. The rotation of rice-potato produced the highest biomass production, followed by ryegrass, rapeseed, and Chinese milk vetch (Figure 1). However, considering the harvest of RR for pasture and the burned straw of ROM, the amount of plant residues returned to the soil was limited. Therefore, only three rotations of RP, RC, and ROF had the organic material returned. RP had the highest value of returned organic material, but the yield improvement was limited. Ghoshal and Singh [47] suggested that crop biomass and grain yield were improved when straw was returned. However, Henderson [51] remarked that most of the studies did not provide an insight into how the procedure influenced crop yield. The results obtained were often site- and year-specific and often contradictory and inconclusive due to variability in soil type, cropping systems, and climate [52, 53]. Therefore, further research is needed to investigate the influence of organic return on rice yield in different paddy-upland rotations.

### 4.2. Paddy-Upland Crop Rotation Effects on Soil Quality Attributes

In paddy-upland crop rotations, soil puddling in advance of transplanting can foster high productivity [54]. This procedure involves plowing the soil when wet, puddling it, and keeping the area flooded for the duration of rice growth. Puddling breaks down and disperses soil aggregates into microaggregates and individual particles [12]. However, continuous use of this method of rice cropping will destroy soil structure and create a poor physical condition [13, 14]. In this study, RC and RP improved the soil MWD compared to CK. In addition, RC and ROF increased the soil bulk density compared with CK (Table 3). These results suggest that rice rotated with milk vetch might improve the paddy soil structure by increasing the soil MWD. 

Significant soil chemical quality diversity was found among rotations. Soil pH value was acid in the paddy field, which was consistent in our results. Application of ROM increased the pH value as well as available K compared with CK. This might be due to the use of rapeseed straw burned into ash and applied as fertilizer. Biomass ash is considered as a potassium fertilizer in China. Furthermore, improved TN and SOM were found in RC and RP. The positive relationship between yield and TN and SOM might provide the evidence for the positive effect of RC rotation on rice yield. 

Soil quality is different for different crop species [55–57] as well as the proper management of crop residues in terms of improving soil organic matter dynamics and nutrient cycling [58, 59]. In our experiment, soil quality was influenced by both crop species and residue management, and the integrated results were shown by soil quality indicators. Green manure crop (milk vetch) cultivation of upland plant species during the fallow seasons in the monorice cultivation system was found to improve soil fertility because of its higher nitrogen fixing capabilities and rapid biomass accumulation characteristics [49, 50] We found that MWD, TN, CEC, SOM, DOC, and MBC improved in rice-milk vetch rotations compared with CK. However, increased bulk density was also found in the rice-milk vetch rotation; this has a generally negative effect on soil quality. Rapeseed and winter rye can be sown after rice and harvested before rice transplanting. These systems can maximize benefits of the rotation as well as availability and resources [60]. *Brassica* species are important oil seed crops in China and can aid in controlling pests and weeds because of the allelochemicals they release. In this study, two different rapeseed agronomic practices were introduced in terms of different harvest dates. Unlike the regular harvest practice for oil seed, the rapeseed was harvested artificially at flowering, when its glucosinolate content is relatively high [61, 62]. In addition, rapeseed also has potential for use as a green manure crop, which may prevent soil erosion and reclaim leachable nutrients [63, 64]. We found that ROM and ROF had the most soil CEC, TP, and available K in 10–20 cm depth (Table 4) and that ROF has the highest value of all the rotations in MBC in 10–20 cm depth (Table 5).

### 4.3. Paddy-Upland Crop Rotations on Soil Bacterial Communities' Diversity

Soil bacterial communities were significantly affected by soil type and plant species as well as environmental factors. As shown in Figure 3, two major separate branches were found among eight rotations: pretransplanting (BR) and postharvest (AR). Within rotations, CK was totally different from the others; it had the lowest similarity value regardless of sample time, with a mean similarity of 62.5% at BR and 71.3% at AR to others. Further, four out of the other five rotations were grouped together with a mean similarity to RP of 69.8% at BR and 75.8% at AR. These results indicate remarkably different long-term paddy-upland rotation effects on soil bacterial community construction. However, further analysis is needed to explore the details of these bacterial communities.

## 5. Conclusions

Significant differences in soil chemicals (i.e., soil pH, TN, CEC, SOM, and MBC), physical properties (soil bulk density), and soil bacterial communities were detected between cropping seasons within the year (rice and upland crops season), irrespective of different winter upland crop species. Rice-Chinese milk vetch and rice-rapeseed rotations improved the soil quality to some extent, which might result in the greatest yield performance in rice-Chinese milk vetch rotations among the tested rotations. Soil bacterial communities in CK and RP were remarkably different from those in the other rotations according to the T-RFLP of 16S rRNA genes.

## Figures and Tables

**Table 1:** Experimental design of six paddy-upland crop rotation systems.

Label	Cropping system	Year of start of experiment	Rice season			Upland crop season		Herbicides	Annual N-P-K input Kg/ha	
			Sowing	TP	Harvest	Sowing	Harvest		Rice	Upland crop season
CK	Rice-fallow phase	Continues	Early May	Early June	Late October	—	—	Yes		No
RR	Rice-ryegrass	2001				November	Harvest 2-3 times	Yes	^ b^2001–2005: 180-50-180	No
RP	Rice-potato, with rice straw as mulch	2001				December	March	No		No
RC	Rice-Chinese milk vetch	2001				November	—	No	2005–by now: 120-50-120	No
ROM	Rice-oilseed rape with burned straw return after harvest	2001				November	April	No		^ c^20-20-20
^ a^ROF	Rice-oilseed rape with fresh straw return as green manure at flowering	2003				November	March	No		No

^a^Oilseed rape was considered as manure crops but economic crops in ROF. This is a new cropping rotation under evaluating.

^b^Fertilizer input was reduced due to the lodging problem of rice season in RP and RC treatment.

^c^Fertilizer applied at bolting stage for oilseed rape.

**Table 2:** General soil properties before the experiment started in 2001 (0–20 cm soil depth).

Treatment	pH	SOM	TN	TP	^ a^Available K	Bulk density
		(g/kg)	(g/kg)	(g/kg)	(g/kg)	(g/cm^3^)
CK	6.51	26.90	2.53	0.62	0.22	1.17
RP	6.69	24.50	2.49	0.65	0.24	1.21
RR	6.82	25.60	2.50	0.63	0.23	1.12
RC	6.62	29.10	2.46	0.66	0.23	1.24
ROM	6.43	27.50	2.69	0.61	0.22	1.11
ROF	—	—	—	—	—	—
Means	6.61	26.72	2.53	0.64	0.23	1.17
^ b^CV%	2.30	4.81	3.32	3.08	3.61	4.85

^a^Available K was extracted with 1 mol NH_4_AC.

^b^C.V. is coefficient of variation.

Abbreviations: CK: rice-fallow phase; RR: rice-ryegrass; RP: rice-potato, with rice straw as mulch; RC: rice-Chinese milk vetch; ROM: rice-oilseed rape with burned straw return after harvest; ROF: rice-oilseed rape with fresh straw return as mulch at flowering.

**Table 3:** Soil physical properties in 0–20 cm depth.

Rotation	^ a^Sand	Slit	Clay	MWD	Bulk density
	(g/kg)	(g/kg)	(g/kg)	(mm)	(g/cm^3^)
CK	40.9b	650.9	300.0b	0.07b	1.20b
RC	89.4a	574.5	336.1b	0.11a	1.39a
RR	51.4b	559.2	389.4a	0.08b	1.26ab
RP	76.2a	586.2	337.5b	0.10a	1.21b
ROM	54.6b	551.5	393.9a	0.08b	1.21b
ROF	42.6b	531.4	426.0a	0.06b	1.35a

^a^Sand 2–0.05 mm, silt 0.05–0.002 mm, clay < 0.002 mm.

Means on the same column and for the same sampling time followed by the same letter (or none) are not significantly different at *P* < 0.05 by Tukey Test. Abbreviations: CK: rice-fallow phase; RR: rice-ryegrass; RP: rice-potato, with rice straw as mulch; RC: rice-Chinese milk vetch; ROM: rice-oilseed rape with burned straw return after harvest; ROF: rice-oilseed rape with fresh straw return as mulch at flowering.

**Table 4:** Soil chemical properties in 0–10 cm depth.

Depth	Rotation	pH	Total N	Total P	Available K	CEC
		(1: 2.5 H_2_O)	(g/kg)	(g/kg)	(g/kg)	(Cmol/kg)
0–10 cm	CK	5.78a	2.46b	0.53b	0.25b	10.73b
	RC	5.48b	2.92a	0.59b	0.28ab	12.23a
	ROF	5.21c	2.82a	0.70a	0.30a	10.97b
	ROM	5.64a	2.56b	0.59b	0.33a	11.92a
	RP	5.15c	2.96a	0.72a	0.31a	10.80b
	RR	5.40b	2.67b	0.67a	0.24b	11.23b

10–20 cm	CK	5.86b	2.36b	0.47	0.22b	10.26c
	RC	5.82b	2.61ab	0.49	0.23b	13.50a
	ROF	6.00a	2.52b	0.54	0.23b	11.41b
	ROM	5.96a	2.41b	0.48	0.30a	11.43b
	RP	5.84b	2.70a	0.53	0.25b	11.01b
	RR	5.89b	2.48b	0.54	0.21b	10.34c

Comparison of depth	0–10 cm	5.44b	2.73a	0.63a	0.29	11.31
	10–20 cm	5.90a	2.51b	0.51b	0.24	11.33

ANOVA	Rotation	∗∗	∗∗	∗∗	∗∗	∗∗
	Depth	∗∗	∗∗	∗∗	ns	ns
	R × D	∗	∗	∗	∗∗	∗∗

Means on the same column and for the same sampling time followed by the same letter (or none) are not significantly different at *P* < 0.05 by Tukey Test. ns: not significant; *P < 0.05; **P < 0.01. Abbreviations: CK: rice-fallow phase; RR: rice-ryegrass; RP: rice-potato, with rice straw as mulch; RC: rice-Chinese milk vetch; ROM: rice-oilseed rape with burned straw return after harvest; ROF: rice-oilseed rape with fresh straw return as mulch at flowering.

**Table 5:** Soil organic carbon content, dissolved organic content, and soil microbial organic content in 0–10 and 10–20 cm depth

Rotation	SOM	DOC	MBC	SOM	DOC	MBC
	(g/kg)	(g/kg)	(g/kg)	(g/kg)	(mg/kg)	(g/kg)
	0–10 cm			10–20 cm		

CK	21.6b	0.08b	0.76b	22.2ab	0.07b	0.64b
RC	25.8a	0.16a	1.08a	21.4b	0.13a	1.11a
ROF	20.3bc	0.15a	1.22a	16.6c	0.12a	1.08a
ROM	18.9c	0.09b	0.51b	17.1c	0.15a	0.44b
RP	23.7a	0.20a	0.57b	23.4a	0.08b	0.60b
RR	21.3b	0.19a	1.09a	18.6c	0.14a	1.04a

Comparison of depth						
0–10 cm	21.93a	0.15	0.87			
10–20 cm	19.88b	0.11	0.82			

ANOVA						
Rotation	∗∗	∗∗	∗∗			
Depth	∗∗	ns	ns			
R × D	∗∗	∗∗	∗∗			

Means on the same column and for the same sampling time followed by the same letter (or none) are not significantly different at *P* < 0.05 by Tukey Test; ns: not significant; *P < 0.05; **P < 0.01. Abbreviations: CK: rice-fallow phase; RR: rice-ryegrass; RP: rice-potato, with rice straw as mulch; RC: rice-Chinese milk vetch; ROM: rice-oilseed rape with burned straw return after harvest; ROF: rice-oilseed rape with fresh straw return as mulch at flowering.
